# Biofloc Microbiome With Bioremediation and Health Benefits

**DOI:** 10.3389/fmicb.2021.741164

**Published:** 2021-11-29

**Authors:** Vikash Kumar, Suvra Roy, Bijay Kumar Behera, Himanshu Sekhar Swain, Basanta Kumar Das

**Affiliations:** ^1^Aquatic Environmental Biotechnology and Nanotechnology (AEBN) Division, ICAR-Central Inland Fisheries Research Institute (CIFRI), Barrackpore, India; ^2^Fisheries Enhancement and Management (FEM) Division, ICAR-Central Inland Fisheries Research Institute (CIFRI), Barrackpore, India; ^3^ICAR-Central Inland Fisheries Research Institute (CIFRI), Barrackpore, India

**Keywords:** bioremediation, pathogenic microbes, heterotrophic microbes, biofloc system, host immunity

## Abstract

The biofloc system has recently attracted great attention as a cost-effective, sustainable, and environmentally friendly technology and expected to contribute toward human food security (Zero Hunger SDG 2). It is also expected that this endeavor can be adopted widely because of its characteristics of zero water exchange and reduced artificial feeding features. In the biofloc system, the flocs which are generally formed by aggregation of heterotrophic microorganisms, serve as natural bioremediation candidates. These microbes effectively maintain water quality by utilizing the nutrient wastes, mostly originated from digested, unconsumed, and metabolic processes of feed. Additionally, the flocs are important sources of nutrients, mainly a protein source, and when these are consumed by aquaculture animals they improve the growth performance, immunity, and disease tolerance of host against pathogenic microbial infection. Here in this review, we focus on recent advances that could provide a mechanistic insight on how the microbial community developed in the biofloc system helps in the bioremediation process and enhances the overall health of the host. We have also tried to address the possible role of these microbial communities against growth and virulence of pathogenic microbes.

## Introduction

Aquaculture, the farming of aquatic animals and plants, continues to dominate the food producing sector in the world (FAO, 2019). The global aquaculture production increased to 4.9% as compared to 2016 and reached to 111.9 million tons in 2017. The aquaculture share in total global aquatic animal production including both capture and aquaculture has risen sharply from 25.7% in 2000 to 46.4% in 2017, with an average annual growth rate of 4.8% during 2011–2017 (Kumar, 2020; Roy, 2020; Tacon, 2020). The 10 top aquaculture producing countries including China, India, Indonesia, Vietnam, Bangladesh, Egypt, Norway, Chile, Myanmar, and Thailand, contribute over 88% by quantity of global aquaculture production in 2017. Interestingly, the aquaculture industry is playing a major role in economic development and together with capture fisheries it supports the livelihood of more than 10% of the world population (Kumar et al., 2021b). Moreover, as the global human population continues to expand at a high rate and is expected to reach over 9 billion by 2030, the aquaculture industry could be crucial for food and nutritional security with high-quality animal protein in both inland and coastal regions, and providing livelihood and income source generation to millions of people (Roy et al., 2019, 2020; Ngasotter et al., 2020). However, due to the global demand increase, the pressure for intensification and further expansion of culture systems has created many problems including scarcity of natural resources, increased environmental pollution and losses due to disease outbreak. Disease outbreaks caused by bacterial infections, considered as the primary cause of production loss in fish farming, have moved to the forefront in recent years and brought socio-economic and environmental unsustainability to the aquaculture industry (Costa-Pierce et al., 2010; Verdegem, 2013; Kumar V. et al., 2018, Kumar et al., 2020a). The economic losses in the aquaculture sector from disease outbreak has been calculated by FAO to be over US$9 billion per year, that is approximately 15% of world aquaculture fish and shellfish production, by value (Kumar et al., 2019a, b; Tran et al., 2020). Additionally, the fish feed, the prices vary from a few hundred dollars a ton to more than US $1000 a ton depending on the species being fed, is the major operational cost for most fish farms accounting for 50–70% of the variable cost (FAO, 2019). In addition, in developing countries like Indonesia, Vietnam, or Bangladesh, the commercial feed is simply beyond the reach of most marginal and landless farmers, limiting their ability to intensify aquaculture production. In this context, development of culture protocol/technology that can reduce the input cost and enhance the immunity and disease tolerance of cultured animals seems to be a preferable alternative for aquaculture systems.

**FIGURE 1:**
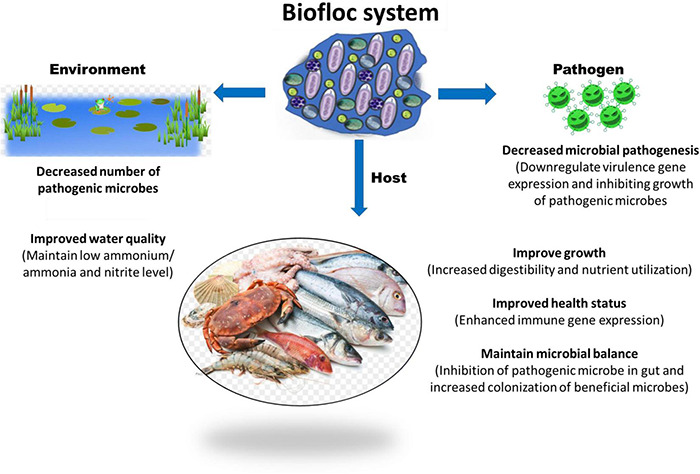
Potential role of biofloc system in host, pathogen, and environment in a culture facility.

In recent years, growing aquaculture species in the biofloc system is becoming more popular and has shown promising results in improving water quality, fish health, and production. This technology could become essential not only to cover the growing demand for dietary animal proteins but also for the water scarcity, environmental issues, and animal health and disease. The biofloc system’s basic principle is to recycle and transform waste and excessive nutrients, in particular inorganic nitrogen (NH_3_-N and NO_2_-N), generated from feces and uneaten feed into microbial biomass. These biomasses are generally high in protein content and utilized by cultured animals *in situ* or if harvested they could be processed and used as a nutrient source for feeds. This is achieved by steering the carbon and nitrogen ratio (*C*/*N* ratio) through modification of feed carbohydrate content or carbon source addition in water (Avnimelech, 1999; Kuhn et al., 2009; Crab et al., 2012; Ekasari et al., 2014; Fatimah et al., 2019; Hostins et al., 2019). For instance, Schneider et al. (2005) reported that if the *C*:*N* ratio is maintained between 10 and 15:1, the organic nitrogenous ammonium waste are converted into bacterial biomass (Schneider et al., 2005). In another study, Crab et al. (2007) noted that the use of biofloc system in intensive tilapia culture significantly improved the nitrogen recovery from 23 to 43% and non-toxic levels of ammonia/ammonium concentration could be maintained, without water exchange (Crab et al., 2007). There are also few reports that suggest that biofloc contains microbe-associated molecular pattern (MAMP) and microbially bioactive components such as carotenoids, vitamins, glutathione, antioxidants, and minerals, which nutritionally modulate the fish health and immune response and resulted in better growth performance and increased resistance against microbial pathogens (Xu and Pan, 2013; Hostins et al., 2019; Kumar et al., 2020b) (Figure 1). Since the biofloc system has several beneficial effects that contribute to the maintenance of optimum water quality in the culture system and improvement of feed utilization and nutrition of the cultured animals, the technology has a wide range of acceptability across several aquaculture producing countries. However, still refinements of the biofloc culture protocol in terms of growth and nutrition requirements of aquaculture species is required. In addition, more importantly, information on biofloc derived microbes and how these microbial origins affect the external aquatic pathogens and beneficially improve the aquatic environment and host survival is needed for better understanding and scientific application of the biofloc system. Hence, in this review at first an overview of the current knowledge on the effect of the biofloc system microbial community on aquatic environment and host is given. Later, the possible role of these microbes on the activity and virulence of pathogenic microorganisms with respect to aquaculture are summarized.

## Development of Standard Biofloc System: Considerations for Optimal Microbial Consortia Throughout the Culture Cycle

The biofloc system is a relatively new aquaculture technology that allows high-density culture at a limited or zero water exchange facility. Interestingly, once the *C*/*N* ratio reaches between 10 and 15:1, by addition of exogenous carbon source, the available microbe in the system utilizes the accumulated nitrogenous wastes originated from unconsumed feed and animal excretion, including fecal materials and metabolic products and accelerates the growth of microbial communities that conglomerate together and produce flocs (Ju et al., 2008; de Jesús Becerra-Dorame et al., 2014). The potential of this system in increasing the resource utilization efficiency has raised the attention for both research and application during the past decade. As a consequence, more beneficial effects of the biofloc system have been discovered including the nutritional properties, exogenous digestive enzymes contribution, potential control of pathogens, and immunostimulatory effects (Betanzo-Torres et al., 2020; Vyas, 2020; El-Sayed, 2021). Few reports have also suggested that the biofloc formation is mediated by one of the microbial phenotypes, which are involved in the production of small membrane diffusible metabolites (Chong et al., 2012; Hawver et al., 2016). Therefore, it becomes very intriguing to investigate and characterize the biofloc microbiome stem from their ability to maintain water quality and confer immunostimulatory effect on the cultured animals.

To develop a biofloc, at first the tanks (might be circular or rectangular, however, circular tanks are preferred) were filled with water, and nitrogenous material (fish feed and urea fertilizer) and a carbon source (molasses, wheat flour, or starch, about 0.7% of feed) were added to the water (Table 1). In some cases, soil clay particles, after mixing thoroughly and filtering with a sieve, were also added to the tanks, it helps in biofloc formation and further mass continuity. Afterward, the primary inoculum of the microbial biomass and necessary elements, including proper aeration, were added to the system, it improves the microbial flocs formation in the new culture system (Zemor et al., 2019). As a standard, 20 g of clay, 10 mg of ammonium sulfate, and 200 mg of carbonaceous organic matter such as molasses can stimulate biofloc formation in 1 L of water. The presence of carbonated organic matter enables heterotrophic bacteria to become more active than other bacteria, and they remove nitrogen and carbon from water by an absorption process and produce microbial biomass/flocs. These biomasses were subsequently combined/fed by other organisms (*viz*., algae, detritus, ciliates, or yeast) and collectively form biofloc in the culture system (Khanjani et al., 2017). During the biofloc formation, algae were first developed and form foam, and eventually microbial biomass. The development of a brown state in culture tanks indicates the presence and activity of heterotrophic bacteria. Moreover, once the experimental animals were added to the biofloc system, the physicochemical parameters (temperature, oxygen, pH, alkalinity, total nitrogen, ammonium, nitrite, and nitrate) should be measured weekly. In the case of any deviation from standard water quality parameters, subsequently appropriate responses including water exchange, stopped artificial feeding, etc. should be adopted quickly (Avnimelech, 1999; Khanjani and Sharifinia, 2020). For instance, if the ammonia levels were high, then input carbon source might be increased while decreasing the feed content. Similarly, if the nitrite level is high, then increase the carbon input and check oxygenation and sludge collection. Moreover, if microbial biomass is low, add carbon source and if the volume of the biofloc is too high, then do a partial water exchange.

**TABLE 1:** The use of different carbon sources and *C*/*N* ratio for development of stable biofloc culture system.

**Species**	**Carbon sources**	***C*/*N* ratio**	**References**
*Macrobrachium rosenbergii*	Glucose, glycerol and acetate	10	Crab et al., 2010
*Litopenaeus vannamei*	Dextrose	First 3 days – 20; 4–30th days- 6	Suita et al., 2015
	60% molasses + 20% corn flour + 20% wheat bran	16	Wang et al., 2016
	Molasses + dextrose + rice flour	First 5 days – 15; 6–70th days- 6	Serra et al., 2015
	Glucose	15	Kumar et al., 2020b
	Molasses + wheat flour + starch	15	Khanjani et al., 2017
	Molasses + palm sap	20	Abbaszadeh et al., 2019a, b
	Maida flour, wheat flour, gram flour, millet flour, rice flour, corn flour, molasses and multigrain flour	10–20	Panigrahi et al., 2019b
	Molasses	15	Panigrahi et al., 2019a
	Molasses, tapioca, tapioca by-product, and rice bran	15	Ekasari et al., 2014
	Molasses	12, 15, 18	Xu et al., 2018
*Penaeus monodon*	Tapioca powder	12	Arnold et al., 2009
*Litopenaeus vannamei* and *Penaeus monodon*	Molasses	–	Burford et al., 2004
*Litopenaeus vannamei* and *Macrobrachium rosenbergii*	Starch	10, 15, and 20	Asaduzzaman et al., 2008
*Farfantepenaeus brasiliensis*, and *Farfantepenaeus duorarum*	Wheat flour + molasses	20	Emerenciano et al., 2012
*Farfantepenaeus paulensis*	Wheat bran + molasses	20	Emerenciano et al., 2011
*Farfantepenaeus brasiliensis*	Wheat bran + molasses	20	Emerenciano et al., 2012
*Oreochromis niloticus*	Wheat flour	8–11	Azim and Little, 2008
	Wheat flour and molasses	15	Mirzakhani et al., 2019
Tilapia	Cellulose	11–16	Avnimelech, 2009

From the studies mentioned in the above paragraphs, it is clear that the bacterial population, i.e., heterotrophic bacteria, are the predominant group of biofloc microbiota; however, fungi, algae (dinoflagellates and diatoms), flagellates, rotifers, ciliates, and detritus also constitute the microbial components (Ju et al., 2008; Kim et al., 2014; Kasan et al., 2017; Tepaamorndech et al., 2020). For instance, Cardona et al. (2016) analyzed the biofloc system water samples used for culture of *Litopenaeus stylirostris* using 16S rRNA amplicon sequencing. The results showed that bacteria taxa belonging to Proteobacteria, Bacteroidetes, and Cyanobacteria groups have the highest relative abundance (Cardona et al., 2016). Later, Tepaamorndech et al. (2020) performed 16S rRNA amplicon sequencing and shotgun metagenomic analysis to characterize the complex of bacterial communities in the biofloc system culturing *Litopenaeus vannamei*. The analysis revealed that 90% biofloc microbial population comprised of *Vibrio* sp., while *Bacillus*, *Lactobacillus*, *Pseudoalteromonas*, *Clostridium*, *Shewanella*, *Acinetobacter*, *Photobacterium*, *Alteromonas*, *Marinifilum*, and *Pseudomonas* were also identified. In another study, Wei et al. (2020) investigated the microbial communities in the biofloc ecosystem with high-throughput sequencing and quantified the 16S rRNA gene. Findings suggest that bacterial groups belonging to *Flavobacteriaceae* (e.g., *Marivita, Ruegeria*, and *Maribacter*) and *Rhodobacteraceae* were the key bacterial taxa in the biofloc system (Wei et al., 2020). Recently, Islam et al. (2021) evaluated the microbial community structure in biofloc culturing *Macrobrachium rosenbergii*. It was observed that biofloc systems maintained in 15–20:1 *C*:*N* ratio have a higher count of *Lactobacillus* spp. followed by *Enterococcus* spp. (Islam et al., 2021). In another study, Meenakshisundaram et al. (2021) characterize the microbial composition of grown-out biofloc used for culture of genetically improved farmed tilapia (GIFT). The metagenomic profile analyzed through Illumina Nextseq500 platform shotgun sequencing showed that microbial composition in biofloc includes 70.80% bacteria, 5.08% eukarya, 0.62% archaea, 0.16% virus and 23.35% are unclassified. Further classification reveals that abundant genus in biofloc microbiome are *Proteobacteria* and *Caldilinea aerophila* (Meenakshisundaram et al., 2021). Taking together, these studies highlight that dominant microbiota in biofloc system, i.e., *Lactobacillus*, *Bacillus*, and *Vibrio* along with other bacterial groups, e.g., *Halomonas*, *Providencia*, *Nitratireductor*, *Pseudoalteromonas*, etc. might be responsible for inducing beneficial effect on host, environment, and pathogenic microbes, respectively.

Moreover, it is important to realize that quorum sensing (QS), a bacterial intercommunication system that controls the expression of numerous genes, regulates the activities of a large group of bacterial cells (Fuqua et al., 1994; Bassler et al., 1997; Xu et al., 2006). A QS system uses small signal molecules called autoinducers (AIs) to control directly or indirectly bacterial bioluminescence, virulence factor expression, biofilm formation, motility, entry into stationary phase, sporulation, and mating (Miller and Bassler, 2001; Schauder and Bassler, 2001). Additionally, the QS mediated formation of extracellular polymeric substances (EPS) matrix leads to transformation of planktonic bacterial cells into sessile mode of growth and form microbial clusters or aggregates (Kumar et al., 2020a, 2021a). In clusters or biofilm mode of life, bacteria play a vital role in removing or converting harmful compounds and is considered as an excellent biosorbent material for the remediation of toxic substances. Additionally, these aggerates are excellent microbial protein sources and help to improve growth, immunity, and survival of consuming host animal against both biotic and abiotic stressors. Interestingly, one recent study has demonstrated that microbial quorum sensing plays important roles in biofloc characteristics and functionality from aquaculture perspective. The QS regulated the biofloc formation, protein contents, total ammonium nitrogen (TAN) removal capacity and growth of cultured African catfish, *Clarias gariepinus* (Fatimah et al., 2019). However, further characterization on dominant microbial species involved in QS-regulated microbial gene expressions will be helpful to find the possibility to modulate QS activity in the biofloc microcommunity. In addition, it will be also useful in identification of microbial phenotypes that are beneficial in aquaculture perspective, such as the excretion of various digestive enzymes that may contribute to the increased food digestibility of fish and production of essential nutrients that could improve the nutritional value of bioflocs.

## Potential Role of Biofloc System-Induced Microbial Community

**FIGURE 2:**
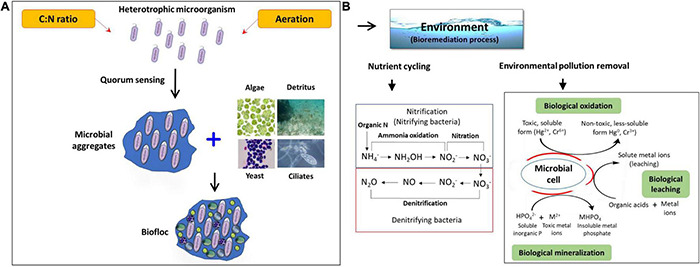
Schematic overview on the possible role of the biofloc microbiome. **(A)** Development of a biofloc system; **(B)** potential role of the biofloc system in the bioremediation process.

The functions of biofloc are strongly related to the interaction of the microbial community in the spatial cohabitation involved in the acquisition of nutrients and the biochemical processes (Ekasari et al., 2014; Bossier and Ekasari, 2017). These communities play an essential role as natural bioremediation candidates in maintenance of water quality and conversion of nitrogenous waste materials. Additionally, they also play a significant part in development of nutrient rich flocs that serve as food sources and contribute in nutrition to support high density growth of aquaculture animals (Figures 1, 2) (Zhao et al., 2014; Zhang et al., 2016).

### Bioremediation Process

Bioremediation is a process of contaminated water or waste treatment into less toxic form using beneficial microorganisms (Das et al., 2019, 2020; Behera et al., 2020a, b). It is achieved by inducing the biological process that leads to reduction, removal, and conversion of the contaminated compounds (Table 2) (Divya et al., 2015; Jasmin et al., 2020). Moreover, the treatment method relies on content and toxicity of contaminants, hydrogeological conditions, ecology of microbial communities, and other temporal and spatial factors (Sarkar et al., 2021). The microbial bioremediation process is among the most preferred ways to remove contamination from a system, as it is cost effective and able to immobilize or destroy the contaminants efficiency (Gadd, 2000). Interestingly, these microbial communities utilize the contaminants as their energy source, e.g., phosphorus and nitrogen forms in contaminants are utilized by microbes as their nutrient source (Jones et al., 2018). It is noteworthy to mention that, it is not always necessary to utilize the existing natural microbial population for bioremediation process, the exogenous microbes or genetically engineered population can also be used for the process (Brim et al., 2003). Based on the carbon source utilization, microorganisms involved in the bioremediation process are mainly classified in two groups, autotrophs and heterotrophs. The autotrophic microbes are able to utilize the inorganic substances and synthesize their own food, which makes the autotrophs a good bioremediator (Musyoka, 2016). The commonly known nitrite-oxidizing and ammonia-oxidizing bacteria are classified under autotrophic microbes (Merchant and Helmann, 2012). The second group under bioremediation microbes are heterotrophic bacteria, that immobilize or destroy the non-living organic material and generate carbon sources to build their own cells. These microbes act as electron donors in catalyzing reactions, during the oxidation of harmful contaminants. Moreover, unlike autotrophs, the heterotrophic bacteria contribute comparatively less in the process of nitrification and denitrification, however, they breakdown the organic waste including feces, uneaten feed, and dead materials and transfer the nitrogenous ammonia into non-harmful products known as microbial aggregates or mass (Ebeling et al., 2006).

**TABLE 2:** The list of microorganisms reported to be involved in the natural bioremediation process, growth, immunity, and disease tolerance of aquaculture animals.

**Microbial species**	**Target system**	**Bioremediation process**	**Growth performance**	**Immune response**	**Disease resistance/antimicrobial activity**	**References**
**Bacteria**						
*Bacillus pumilus* and *Lactobacillus delbrueckii*	Biofloc water	Total ammonia nitrogen (TAN) concentration (−) after 7th week in common carp culture system	Weight gained per day (WGD), specific growth rate (SGR) (+), and Feed conversion rate (FCR) (−)	Lysozyme, respiratory burst and myeloperoxidase activity (+)	Survival against *Aeromonas hydrophila* challenge (+)	Dash et al., 2018
*Aeromonas salmonicida*, *Aeromonas hydrophila* and *Pseudomonas aeruginosa*	Biofloc water	Ammonium 96%, nitrite 37.5% and nitrate 62% (−) in 105 days culture period	–	–	–	Manan et al., 2017
*Bacillus licheniformis*	Biofloc water	–	–	Hemocytes count and total protein content (+)	*In vitro* inhibitory activity (+) and *in vivo* count of *Vibrio alginolyticus* (−)	Ferreira et al., 2015
*Bacillus* sp.	Pond wastewater	Ammonium, nitrite and nitrate (−) in 4 days period	–	–	–	Naderi Samani et al., 2016
*Bacillus vietnamensis* and *Gordonia bronchialis*	Pond wastewater	Total ammonia nitrogen (TAN) and nitrite concentration (−) in 5 days	–	–	–	Muthukrishnan et al., 2015
*Marichromatium gracile* YL28	Pond wastewater	Nitrite removal 99.96% and ammonium assimilation 95.6% from aquaculture pond wastewater in 7 days	–	–	–	Zhu et al., 2019
*Bacillus* sp. mixture	Shrimp culture water	Total ammonia nitrogen, nitrite and nitrate level (−) in 8 weeks *Litopenaeus vannamei* culture	Final weight, weight gain, specific growth rate (SGR) (+) and food conversion ratio (FCR) (−) of cultured animals	Expression of prophenoloxidase (proPO), peroxinectin (PE), lipopolysaccharide- and β-1,3-glucan- binding protein (LGBP) and serine protein (SP) (+)	Survival (80%) (+) as compared to control (40%) against *Vibrio harveyi* infection	Zokaeifar et al., 2014
*Bacillus* sp.	Prawn culture water	Ammonium and nitrite levels (−) in *Macrobrachium rosenbergii* culture after 60 days	Specific growth rate (SGR) (+) and food conversion ratio (FCR) (−) of cultured animals	Total haemocyte count (THC), phenoloxidase (PO) and respiratory burst activity (+)	–	Mujeeb Rahiman et al., 2010
*Bacillus amyloliquefaciens*	Sewage water	Total ammonia nitrogen (TAN) 93% (−) within 24 h	–	–	–	Yu et al., 2012
*Streptococcus, Staphylococcus, Bacillus, Neisseria* sp.	Sewage water		Develops biofloc that might help in improved growth performance.			Kurniawan et al., 2020
*Bacillus* sp., *Lactococcus, Rhodococcus, Kocuria, Pseudomonas.*			Enhanced growth performance	Immune response (+)		
*Nitrospira, Rhodobacter.*		Actively involved in nitrification and denitrification process				
*Sphingomonas, Burkholderia*, and *Acinetobacter.*		Maintain optimum water quality by degradation of organic matters				
*Bacillus subtilis, Bacillus mycoides*, and *Bacillus licheniformis*	Recirculatory system water	Ammonium, nitrite, nitrate and phosphate levels (−) in recirculation tanks	–	–	*In vitro* antimicrobial activity against *Aeromonas hydrophila* (+)	Lalloo et al., 2007
*Bacillus subtilis* and *Bacillus megaterium*	Recirculatory system water	Total ammonia nitrogen and chemical oxygen demand (COD) (−) in red parrot fish recirculation tanks	Weight gain (WG) (+) in treatment as compared to control	–	–	Chen and Chen, 2001
**Bacteria + yeast combination**						
*Pseudomonas stutzeri* LZX301 and *Candida tropicalis* HH8	Biofloc water	Nitrite removal 59.33% and ammonium assimilation 44.87% from culture water in initial 11 days	–	–	–	Gao et al., 2019
*Nitrobacter*, yeast and *Bacillus subtilis*	Pond wastewater	Total ammonium nitrogen 99.74% and 62.78% total phosphorus (−) in brackish aquaculture wastewater	–	–	–	Mohamad et al., 2017
**Bacteria + microalgae combination**						
*Lactiplantibacillus plantarum* and *Schizochytrium* sp.	Biofloc water	Ammonium and nitrite concentration (−), while stabilizing nitrate value in 44 days culture period	–	–	–	Pacheco-Vega et al., 2018

*(+) increased; (−) decreased.*

Interestingly, a biofloc system promotes the growth of both autotrophic and heterotrophic microbes (Manan et al., 2017; Pacheco-Vega et al., 2018). However, bioflocs contain a high number of heterotrophic beneficial microbial communities, including *Bacillus*, *Acinetobacter*, *Sphingomonas*, *Pseudomonas*, *Rhodopseudomonas*, *Micrococcus, Nitrosomonas*, *Nitrospira*, *Nitrobacter*, *Cellulomonas*, and yeast. These microorganisms act as potential bioremediation agents in biofloc culture systems, leading to improving water quality, growth performance, and health of cultured aquatic animals (Thomas et al., 1992; Monroy-Dosta et al., 2013; Das and Dash, 2014; Adel et al., 2017) (Table 2 and Figure 2). The build-up of particulate and dissolved organic matter is a common phenomenon observed in biofloc systems, however, high levels of heterotrophic microbes efficiently minimize the organic nitrogen and carbon levels in the system. These heterotrophic microbes, as potential bioremediators, produce diverse metabolic enzymes which assist in safe removal of contaminants either by converting to safer or less toxic substances or direct destruction (Dash and Das, 2012). For instance, Manan et al. (2017) carried out an experiment to determine the role of aggregating biofloc in the bioremediation process including degradation and decomposition of organic matter. The results showed that heterotrophic bacteria identified from *Aeromonas* (*Aeromonas salmonicida* and *Aeromonas hydrophila*) and *Pseudomonas* family (*Pseudomonas aeruginosa*) consumed the bottom organic matter of shrimp (*L. vannamei*) culture biofloc tanks. In addition, after converting these bottom wastes through chemical processes, they help in the production of high protein flocs that are utilized by the cultured shrimp (Manan et al., 2017). It is important to mention that *Aeromonas* and *Pseudomonas* sp. could be pathogenic to shrimp species (Ramalingam and Ramarani, 2007; Zhou et al., 2019); hence, it becomes important to validate the pathogenicity of these bacterial strains before concluding it as beneficial microbes. In another study, Hostins et al. (2019) designed an experiment to investigate the effect of autotrophic (with or without probiotics) and heterotrophic biofloc (with or without probiotics) cultured with *L. vannamei* against AHPND bacterial strain. The results showed that heterotrophic biofloc (with and without probiotics) and autotrophic biofloc (with probiotics) can decrease the impact of AHPND-causing *Vibrio parahaemolyticus*. However, in heterotrophic biofloc, there was significant improvement in water quality and *L. vannamei* showed the highest survival with and without probiotic supplementation, when challenged in the presence of their respective biofloc suspensions (Hostins et al., 2019). There were also few reports that suggest that association of bacteria (*Pseudomonas stutzeri* LZX301/Nitrobacter/*Bacillus subtilis*) with yeast (*Candida tropicalis* HH8) or microalgae (*Schizochytrium* sp.) is effective in maintaining optimum water quality by decreasing the total ammonium nitrogen, nitrite, and nitrate concentration (Mohamad et al., 2017; Pacheco-Vega et al., 2018; Gao et al., 2019). Recently, Kurniawan et al. (2020) investigated the diversity and abundance of biofloc forming bacteria in the river waters using 16S rDNA sequencing method. The analysis revealed that seven bacterial phyla including *Proteobacteria*, *Cyanobacteria*, *Verrucomicrobia*, *Actinobacteria*, *Bacteriodetes*, *Chloroflex*, and *Planctomycetes* and 14 bacterial genera *Streptococcus, Staphylococcus, Bacillus, Neisseria* sp., *Bacillus* sp., *Lactococcus, Rhodococcus, Kocuria, Pseudomonas, Nitrospira, Rhodobacter, Sphingomonas, Burkholderia*, and *Acinetobacter* have potential biofloc forming abilities (Kurniawan et al., 2020).

### Growth and Immunity

The beneficial effects of bioflocs on growth and immunity of farmed animals have been widely documented. Bioflocs can enhance innate/non-specific immune systems of cultured species through providing a wide range of immunostimulatory effects against microbial infections. The heterotrophic microbial cell walls could contain either lipopolysaccharides, glucans, or peptidoglycans. These microbe-associated molecular patterns (MAMPs) can activate the non-specific immune mechanisms, leading to significant enhanced immune response in farmed species (Aguilera-Rivera et al., 2019; Panigrahi et al., 2019a, 2020). For example, the heterotrophic biofloc reared *L. vannamei* showed enhanced immune response and increased level of total hemocyte count (THC) and prophenoloxidase (ProPO) activity as compared to control animals (Panigrahi et al., 2019b). Bioflocs are also capable of accumulating bacterial compound, namely, poly-β-hydroxybutyrate (PHB), which has been reported to improve growth performance, food digestibility, and development of resistance against bacterial infections in farmed aquatic animals (Kuhn et al., 2009; Khanjani and Sharifinia, 2020). These beneficial microbes can positively modulate the gut microbiota resulting in enhanced growth performance and immune response of the host (Balcázar et al., 2006; Pérez et al., 2010). In addition, the biofloc microbial species contain several nutritional factors and digestive enzymes, e.g., amylase and proteases, which could contribute in the natural digestive process and improve the food digestion and absorption, resulting in efficient utilization of feed and enhanced growth performance of the host (Wang, 2007; Liu et al., 2009). For instance, *Bacillus* sp. has been reported to contribute in host nutrition, especially by supplying vitamins and fatty acids and improve the growth and survival of aquaculture animals (e.g., *Penaeus monodon* postlarvae) in zero water exchange facility (Devaraja et al., 2013; NavinChandran et al., 2014; Kumar et al., 2016). In another study, Zokaeifar et al. (2012, 2014) demonstrated that *Bacillus* sp. significantly enhances the activity of digestive enzyme, growth performance, immunity, and resistance of shrimp toward bacterial infection. These results highlight that beneficial microbe in the system, namely, *Bacillus* sp., could significantly improve the amylase and protease activity and subsequently increase the final weight and weight gain of shrimp juveniles. Interestingly, in a few other studies, *Bacillus* sp. have been demonstrated to stimulate the immune response of *L. vannamei* juveniles, resulting in enhanced disease resistance and survival of shrimp against *Vibrio harveyi* challenge (Zokaeifar et al., 2012, 2014). Later, Nimrat et al. (2012) and Sadat Hoseini Madani et al. (2018) evaluated the role of commercial *Bacillus* sp. application on feed efficiency, growth performance, bacterial number, body composition, water quality, and immune response in *L. vannamei*. The results highlight that administration of *Bacillus* sp. to the experiential units significantly enhances the weight gain %, length gain %, specific growth rate %, average daily gain, and FCR of *L. vannamei* as compared to the control group. The beneficial bacteria also improves the water quality parameters, feed utilization, immune response, and survival of *L. vannamei* postlarvae (Nimrat et al., 2012; Sadat Hoseini Madani et al., 2018). In another study, Bachruddin et al. (2018) demonstrated that white leg shrimp (*L. vannamei*) culture water supplemented with beneficial bacterial species, i.e., *Bacillus* sp., significantly increase the feed utilization and improve the total length, weight gain, FCR, and survival of shrimp species (Bachruddin et al., 2018). Kongnum and Hongpattarakere (2012) and Chai et al. (2016) performed an experiment using indigenous *Bacillus* sp., isolated from wild and healthy shrimp intestine, and investigated its effect on shrimp health. The finding suggests that *Bacillus* sp. significantly improves the growth performance, immune system, and resistance of *L. vannamei* against microbial pathogens (Kongnum and Hongpattarakere, 2012; Chai et al., 2016).

**FIGURE 3:**
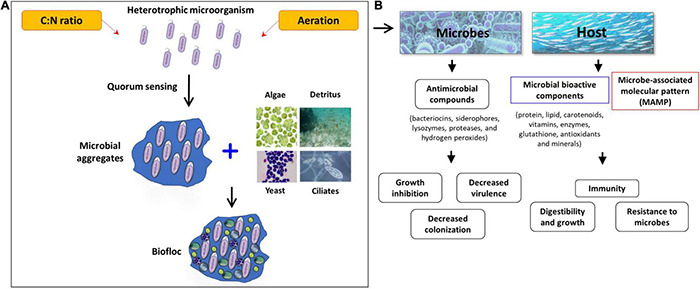
Schematic overview on the possible role of the biofloc microbiome. **(A)** Development of a biofloc system; **(B)** potential role of the biofloc system against pathogenic microbes and in a host.

The bacterial biomass developed as aggregates in the biofloc system can also serve as a nutrient for aquatic animals especially as a protein source and thus improve the growth and overall health of farmed aquatic animals (Table 2 and Figure 3) (Cohen et al., 2005; Azim and Little, 2008; Cardona et al., 2015; Lee et al., 2017). Additionally, these microbes were also demonstrated to produce transduction signaling molecules that have the ability to alert the immune system and protect the host from pathogenic microbial infection (Rendón and Balcazar, 2003; Cerezuela et al., 2013). Hence, administration of these health-benefitting microbes in feed or any sort of incorporation can boost the cellular and humoral component of the innate immune response in both fish and shellfish species (Kuhn et al., 2009; Ahmad et al., 2016; Anand et al., 2017; Kamilya et al., 2017; Kheti et al., 2017; Lee et al., 2017; Fauji et al., 2018; Kumar V.S. et al., 2018). Xu and Pan (2013) reported that shrimp cultured in the biofloc system had higher total hemocyte count and phagocytic activity. Additionally, significantly upregulated total antioxidant and superoxide dismutase capacity and reduced/oxidized glutathione ratio were observed in biofloc cultured shrimp (Xu and Pan, 2013). A study from Kim et al. (2014) suggested that biofloc water contains an abundant number of bacteria biomass and the bacterial cell wall consists of various components such as bacterial peptidoglycan lipopolysaccharide and β-1, 3-glucans, which are potential immunostimulatory agents, stimulating the non-specific immune activity of shrimp. In the experiment, it was demonstrated that the biofloc system significantly enhances shrimp survival, final body weights, and prophenoloxidase (proPO) cascade, which is one of the major innate immune responses in crustaceans, by upregulating prophenoloxidase activation enzyme, masquerade-like proteinase, prophenoloxidase1, prophenoloxidase2, serine proteinase1, and ras-related nuclear protein expression (Kim et al., 2014). Similar findings were also observed in *Labeo rohita* (Ahmad et al., 2016; Kamilya et al., 2017), *Oreochromis niloticus* (Mansour and Esteban, 2017; Mirzakhani et al., 2019; Hwihy et al., 2021; Shourbela et al., 2021), *Cyprinus carpio* (Bakhshi et al., 2018; Aalimahmoudi and Mohammadiazarm, 2019), and *Apostichopus japonicus* (Chen et al., 2018), where animals reared in biofloc water display higher growth performance including improved feed efficiency ratio (FER), specific growth rate (SGR), and feed conversion ratio (FCR). Additionally, the cultured animals have enhanced non-specific immune response, highlighted by significantly increased serum protein, serum albumin, total immunoglobulin, lysozyme, respiratory burst, and myeloperoxidase activity. Taken together, it can be concluded that availability of beneficial microbes in the system can positively improve the growth, immune response, and disease tolerance of cultured animals.

### Disease Resistance

The maintenance of intestinal immunity and metabolic homeostasis is regulated by interaction between host mucosa and the intestinal microbiota. The beneficial microbes were reported to establish a balanced and healthy microbiome within the gastrointestinal tract and minimize harmful bacteria (Kiron, 2012; Gupta et al., 2019). The microbial communities were reported to contain microbe-associated molecular patterns (MAMPs), for instance, bacterial cell wall components like peptidoglycan, lipopolysaccharide, and lipoteichoic acid, which are involved in the modulation of receptor signaling cascades and plays a crucial role in the activation of host immune response and protection from a host from infectious diseases (Cerenius and Soderhall, 2013; Wang and Wang, 2013; Song and Li, 2014; Im et al., 2016). Moreover, there is some evidence that highlights that improved growth performance and non-specific immunity results in development of resistance in cultured animals (Table 2). For example, studies in *O. niloticus* (Elayaraja et al., 2020), *C. gariepinus* (Dauda et al., 2017, 2018; Fauji et al., 2018), *L. rohita* (Kheti et al., 2017), *C. carpio* (Dash et al., 2018), *L. vannamei* (Liu et al., 2017), *Fenneropenaeus indicus* (Megahed et al., 2018), and *M. rosenbergii* (Miao et al., 2017) demonstrated that aquatic animals cultured in biofloc water have significantly enhanced growth and immune response that leads to increased protection against pathogenic microbial infection. Overall, it can be concluded that microbes, mainly heterotrophic bacteria, developed within the biofloc system contribute as bioremediation agents by ensuring optimum water quality and in the process help in the generation of new biomass which are further consumed by cultured animals resulting in improved growth, health and disease tolerance in cultured animals (Liu et al., 2017; Miao et al., 2017; Dash et al., 2018; Megahed et al., 2018; Elayaraja et al., 2020).

## Interaction of Biofloc Derived Microbial Community With Pathogenic Microorganisms

Several researchers have pointed out that biofloc grown aquatic animals have enhanced resistance to pathogenic microbial infections (Figure 3) (Azim and Little, 2008; Crab et al., 2012; Pérez-Rostro et al., 2014; Luis-villaseñor et al., 2016; Anand et al., 2017; Bossier and Ekasari, 2017; Lee et al., 2017; Kasan et al., 2018; Pacheco-Vega et al., 2018; Fatimah et al., 2019; Kumar et al., 2020b). One of the possible scenarios that might be involved behind this induced resistance is improved immunity and health of cultured animals in biofloc as also suggested in an earlier paragraph. It is noted that shrimp in the biofloc system consumes up to 29% flocculating particles of their daily feed intake (Burford et al., 2003), hence the consumed biofloc might nutritionally modulate the health status of shrimp resulting in increased protection of host against microbial diseases. However, another possible mechanism that could also possibly be involved in the protective effect of the biofloc system is intermicrobial interaction that leads to direct growth inhibition or modulation of pathogenic microbe virulence by biofloc derived microbial communities.

The microbes, including both beneficial and pathogenic for cultured species, are ubiquitously present in aquatic environments. The presence of one group in the ecosystem might interact (synergistically or antagonistically) and substantially affects the behavior and abundance of the second group of the microbial community (Matsui et al., 2000; Das et al., 2006, 2014). Bacterial interaction is a common phenomenon that occurs naturally in an aquatic environment. Interestingly, these microbial interactions play a major role in keeping the equilibrium between potentially pathogenic and competing beneficial microorganisms. Nonetheless, the microbial communities composition can be altered by environmental conditions and husbandry practices that stimulate the multiplication of selected bacterial species (Kumar et al., 2016). It is well established that aquatic animals gastrointestinal microbiota can be altered and modified by ingestion of other microorganisms; hence, the microbial manipulation constitutes a promising tool to eliminate or reduce the incidence of opportunistic microbial pathogens (Balcázar et al., 2006). The direct effect, for example, inhibiting the growth and proliferation of other microorganisms, could be the main mode of action of beneficial bacteria that can be observed in cultured systems (Kesarcodi-Watson et al., 2008; Giri et al., 2013; Jiang et al., 2013), and studies have demonstrated that autochthonous microorganisms have significantly high potential because these microbes can easily adapt to the same ecological niche and competitively exclude the pathogenic microbes from the system (Lalloo et al., 2010). Apart from direct inhibition, competition for binding site and nutrition inside the host by adhesion and colonization on the mucosal surfaces are other indirect possible protective mechanisms of beneficial bacteria against pathogenic microbes (Pickard et al., 2017). For instance, study on rainbow trout (*Oncorhynchus mykiss*) illustrated that lactobacilli administration significantly decrease the adhesion and colonization of pathogenic *Carnobacterium piscicola*, *Yersinia ruckeri*, and *A. salmonicida* in intestinal mucus of the host (Balcázar et al., 2007). The beneficial bacteria produce a variety of wide-spectrum chemical compounds including siderophores, bacteriocins, hydrogen peroxides, proteases, and lysozymes in the intestine of the host, thus constituting a barrier against the proliferation of opportunistic pathogenic microbes. Additionally, they produce organic acids that leads to alteration of the intestinal pH due and inhibition of microbial pathogens (Oppegård et al., 2007; Zai et al., 2009; Korkea-aho et al., 2011, 2012; Ström-Bestor and Wiklund, 2011).

Interestingly, there are several studies that have used pathogenic *Listeria monocytogenes* as a model organism, a Gram-positive pathogenic bacterium reported to cause severe problems in the food industry, to study the possible interaction mechanism on growth and virulence of another bacterial community (Zilelidou and Skandamis, 2018). The microbial species, e.g., *Carnobacterium piscicola* (produces bacteriocins) (Buchanan and Bagi, 1997; Nilsson et al., 1999, 2004, 2005; Yamazaki et al., 2003); lactic acid bacteria (LAB)/mainly *Lactobacillus* (pH reduction, lactic acid production) (Callon et al., 2011); *Lactobacillus plantarum* (produces bacteriocins, pH reduction) (Nielsen et al., 2010; Aguilar et al., 2011); *Lactococcus lactis* (produces bacteriocins, pH reduction) (Breidt and Fleming, 1998; Rodríguez et al., 2005; Coelho et al., 2014); *Lactobacillus sakei* (produces bacteriocins) (Vermeiren et al., 2006; Gao et al., 2015; Martinez et al., 2015; Quinto et al., 2016), and *Lactococcus piscium* (Saraoui et al., 2016) are reported to inhibit/reduce the growth of *L. monocytogenes*. There are also few studies that highlight that microbial community groups can antagonistically interact with *L. monocytogenes* and modulate the virulence of pathogens. The microbial species, e.g., *L. lactis* (inhibits biofilm formation) (García-Almendárez et al., 2008; Habimana et al., 2011); *Lactobacillus paracasei, Listeria innocua* (inhibits adherence and biofilm formation) (Bendali et al., 2014; Koo et al., 2014); *Staphylococcus sciuri* (production of siderophores and extracellular polysaccharide) (Leriche et al., 2000), and *Lactobacillus acidophilus* (produce antimicrobial compounds) (Woo and Ahn, 2013) were demonstrated to act antagonistically, while synergistic interaction was observed in *Staphylococcus aureus* and *Flavobacterium* spp. (higher biofilm formation) (Bremer et al., 2001; Rieu et al., 2008). Apart from studies on *L. monocytogenes*, there are also few reports, which indicates the antagonistic properties of bacterial species against pathogenic microbes. For instance, Lalloo et al. (2007) demonstrated that *B. subtilis*, *Bacillus mycoides*, and *Bacillus licheniformis* act antagonistically and significantly inhibit the growth and virulence of *A. hydrophila*.

Moreover, there is not much work done on the effect of biofloc derived microbial communities on the growth and virulence of pathogenic microorganisms. However, few studies like Ferreira et al. (2015) demonstrated that bioflocs are a rich source of beneficial microbial communities, more specifically microbes with probiotics activity. The study showed that Gram-positive bacteria *B. licheniformis*, isolated from biofloc water, have antagonistically inhibited the growth of pathogenic *Vibrio alginolyticus*. Additionally, the *B. licheniformis* modulates growth and immunity and reduces *in vivo V. alginolyticus* count in *L. vannamei* (Ferreira et al., 2015). Recently, Kumar et al. (2020a, b) demonstrated that acute hepatopancreatic necrosis disease (AHPND) causing *V. parahaemolyticus* shift phenotype from virulent state to non-virulent phenotype in a biofloc environment. The results showed that biofloc environment induces adaptive changes in pathogenic *V. parahaemolyticus* strain, observed by downregulation of growth, motility, and virulence related genes. Additionally, the bacterial strain loses the ability to kill the host and when the pathogenic *V. parahaemolyticus* strain added to cultured water it fails to induce significantly high mortality in shrimp species (Kumar et al., 2020a, b, 2021a). Although there was no direct correlation on the possible involvement of biofloc derived microbial communities and observed effect on pathogenic *V. parahaemolyticus*, the results indicate the pathogenic microbe becomes non-virulent in the biofloc system. Taken together, the studies highlight that biofloc associated microbial communities influence the growth and virulence of pathogenic microbes and are involved, at least partially, in providing protection to cultured animals; however, the mechanism by which this phenomenon occurs needs further investigation.

## Commercial Application and Economic Consideration

The adverse climatic condition, namely, droughts, scarcity, and expensive water for the development of aquaculture and the adverse effects of aquaculture effluents on the environment, pollutions, and the spread of infectious diseases have drawn the attention for farm biosecurity and development of alternate technology to reduce the amount of water exchange in the farms (Khanjani and Sharifinia, 2020). The biofloc technology (BFT) is based on the principle of flocculation or co-culture of heterotrophic bacteria and algae within the system (Crab et al., 2007; Ahmad et al., 2017). Interestingly, production in biofloc in the large-scale aquaculture can have environmental benefits in marine and coastal ecosystems. For instance, it can help in minimizing the potential negative effect of artificial commercial feed containing soybean or fish meal on aquaculture wastewater and environmental by serving as a nutrient source in both *in situ* and *ex situ* systems.

The successful demonstration of BFT for several aquaculture species has made this technology a promising endeavor for future fish production. In general, a commercial biofloc system varies in size, between 0.1 and 2 ha and the essential components including aspirators and paddle wheels are installed to aerate and mix the culture water to keep floc particles in suspension. Currently, commercial, large-scale, and small-scale BFT-based fish farms are expanding in a number of countries, especially in Asia (e.g., Indonesia, Malaysia, Thailand, South Korea, China, and India) and the Americas (e.g., United States and Brazil) (Emerenciano et al., 2013). For instance, in Indonesia about 20–25% of shrimp farms have employed the biofloc system, resulting in an average production of more than 20 mt ha^–1^ per cycle in 0.5-ha lined ponds (Crab et al., 2012). Interestingly the lining in ponds, partially or completely lined with high density polyethylene (HDPE) sheets, may affect the overall fish production in the biofloc system. For example, in Malaysia, lower production was achieved (12 mt ha^–1^ per crop) when only pond dikes were lined, while fully lined ponds produced 16.2–22.5 mt ha^–1^ (Bossier and Ekasari, 2017). However, despite the high potential of biofloc technology in aquaculture, the system has some major associated drawbacks. The most important economic problem is the excessive use of energy for continuously high aeration and water mixing. This system also requires regular monitoring, alarms, and emergency power supply. This means that the biofloc systems could increase the operating costs, due to the cost of the aeration system and the carbon source added to the system (Pérez-Rostro et al., 2014). Additionally, after some period the flocs particles tend to become old and increase in size, which might be not acceptable to the cultured fish. Hence, continuous monitoring of flocs volume and particle size is necessary to harness the maximum beneficial effect of the biofloc system. Therefore, it is necessary that economic analyses must be performed before adopting the BFT on commercial scales. In this regard, a species-specific study must be carried out to determine the effectiveness of BFT in aquaculture system.

Although the biofloc system has several attributes that have increased wide adoption for commercial intensive and super-intensive fish culture, data and information on the economy of these systems in Indian major cultured species are almost lacking, or not accessible to the public. Additionally, experience and information generated by private BFT enterprises are generally not open to the public. These companies tend to keep information and their own know how proprietary. Even when the data are available, proper dissemination channels are almost lacking. Hence, studies must be carried out in the future to demonstrate the application of this technology for commercial fish species of India, in order to persuade the farmers to set it up to justify the BFT technology rather than conventional culture methods.

## Conclusion and Future Perspectives

Biofloc technology allows high-density culture and offers the possibility to maintain good water quality with no or minimal water exchange by recycling of nutrient, in particular, nitrogenous waste into microbial biomass that can be utilized *in situ* by the cultured animals. The addition of a carbon source, e.g., molasses or tapioca in *C*:*N* ratio of 12–15:1, promotes aggregation of microbial mass, i.e., biofloc, that helps in natural bioremediation process by converting toxic nutrient from the system and these flocs are subsequently consumed and utilized as a nutrient source for growth, immunity, and developing tolerance against disease by cultured animal. In fact, these microbial masses, which mainly consist of heterotrophic microorganisms, hold enormous potential to promote biofloc development and improve water quality, host immunity and resistance to microbial pathogens. However, still not much work or information is available on the microbial species or diversity responsible for developing flocs and maintaining optimum water quality and health of cultured animals. Hence, isolation of biofloc derived microbial community, mainly heterotrophic microbes, and further characterizing their possible interaction mechanism with environment, host, and pathogenic microbes will open new avenues and will be a promising aquaculture technology for future aquatic environments and pathogen management and possibly result in an overall increase in the aquaculture production with high-density and minimal or no water exchange culture.

Apart from serving as a potential tool to maintain good water quality and the health of the host, the biofloc system derived microbial community can also be useful to manage wastewater by serving as natural bioremediation agents. Wastewater from industry and daily household discharge, which mainly contains nitrogenous compounds, phosphorous, and other dissolved organic carbons, has created havoc in aquatic environments and even destroyed many ecosystems. Interestingly, there is a growing interest in using the microbial community as potential bioremediators to treat the culture water discharge and wastewater. This suggests that identification of a microbial species or consortia from the biofloc system that regulates natural bioremediation processes would be a promising strategy for wastewater treatment. In conclusion, we can say that maintaining a beneficial microbial diversity could be a promising approach to manage wastewater and aquaculture systems.

## Author Contributions

VK: conceptualization, writing original draft, and editing. SR: conceptualization and editing. HSS: editing. BB: conceptualization and supervision. BD: overall supervision.

## Conflict of Interest

The authors declare that the research was conducted in the absence of any commercial or financial relationships that could be construed as a potential conflict of interest.

## Publisher’s Note

All claims expressed in this article are solely those of the authors and do not necessarily represent those of their affiliated organizations, or those of the publisher, the editors and the reviewers. Any product that may be evaluated in this article, or claim that may be made by its manufacturer, is not guaranteed or endorsed by the publisher.
